# Deficit Irrigation with Silicon Application as Strategy to Increase Yield, Photosynthesis and Water Productivity in Lettuce Crops

**DOI:** 10.3390/plants13071029

**Published:** 2024-04-05

**Authors:** Vinícius Villa e Vila, Patricia Angélica Alves Marques, Tamara Maria Gomes, Alan Ferreira Nunes, Victório Goulart Montenegro, Gustavo Soares Wenneck, Laís Barreto Franco

**Affiliations:** 1 Department of Biosystems Engineering, Escola Superior de Agricultura Luiz de Queiroz/ESALQ, University of São Paulo, Padua Dias Avenue, 11, Piracicaba 13418-900, SP, Brazil; paamarques@usp.br (P.A.A.M.); 10699663@usp.br (A.F.N.); vgmontenegro@usp.br (V.G.M.); 2 Department of Biosystems engineering, Faculdade de Zootecnia e Engenharia de Alimentos/FZEA, University of São Paulo, Duque de Caxias, 225, Pirassununga 13635-900, SP, Brazil; tamaragomes@usp.br; 3 Department of Agronomy, State University of Maringa/UEM, Colombo Avenue, 5790, Maringa 87020-900, PR, Brazil; gustavowenneck@gmail.com; 4 Santa Clara Agrociência, Cel. Fernando Ferreira Leite Avenue, 305, Ribeirão Preto 14026-010, SP, Brazil; lais.franco@santaclaraagro.com.br

**Keywords:** biostimulant, irrigation management, *Lactuca sativa* L., water saving, water-use efficiency

## Abstract

In regions where water is a limited resource, lettuce production can be challenging. To address this, water management strategies like deficit irrigation are used to improve water-use efficiency in agriculture. Associating this strategy with silicon (Si) application could help maintain adequate levels of agricultural production even with limited water availability. Two lettuce crop cycles were conducted in a completely randomized design, with a factorial scheme (2 × 3), with three irrigation levels (60%, 80% and 100%) of crop evapotranspiration (ETc), and with and without Si application. To explore their combined effects, morphological, productive, physiological and nutritional parameters were evaluated in the crops. The results showed that deficit irrigation and Si application had a positive interaction: lettuce yield of the treatment with 80% ETc + Si was statistically similar to 100% ETc without Si in the first cycle, and the treatment with 60% ETc + Si was similar to 100% ETc without Si in the second cycle. Photosynthetic rate, stomatal conductance, intercellular CO_2_ concentration, transpiration rate and total chlorophyll content increased under water-stress conditions with Si application; in the first cycle, the treatment with 80% ETc + Si increased by 30.1%, 31.3%, 7.8%, 28.46% and 50.3% compared to the same treatment without Si, respectively. Si application in conditions of water deficit was also beneficial to obtain a cooler canopy temperature and leaves with higher relative water content. In conclusion, we found that Si applications attenuate water deficit effects and provide a strategy to ameliorate the yield and water productivity in lettuce crops, contributing to more sustainable practices in agriculture.

## 1. Introduction

It has been necessary to create strategies that increase the water-use efficiency in irrigated agriculture, and together, increase the vertical production of food for a growing population demand. Deficit irrigation strategy is an agronomic approach for improving water-use efficiency and water management in irrigated agriculture [1]. This strategy consists of performing irrigation with an amount of water below the amount demanded by the crops, which may be during certain periods of growth, such as initial stages or low water demand [2], or even throughout the entire cycle [3]. The main advantage of deficit irrigation is water savings, especially in regions with water scarcity or where water is a limited resource [4]. However, it is important to mention that the application of deficit irrigation requires a detailed knowledge of the specific water requirements of the cultivated plants, as well as the environmental conditions of the region. If poorly managed, this technique can lead to excessive water stress, resulting in a significant reduction in crop yields.

Among leafy vegetables, lettuce (*Lactuca sativa* L.) is the most consumed in the world [5]. Demand grows due to the population’s awareness of healthy eating and a better quality of life, as it is a vegetable that presents many minerals, vitamins and bioactive compounds such as carotenoids and phenolic compounds [6]. There are two typical lettuce production systems, hydroponic and traditional soil cultivation [7]; regardless of the production system, it is a demanding crop in terms of irrigation; that is, in conditions of water deficit, it can have a negative impact on its growth and development. The negative effects caused in plants due to water deficit occur in morphological, productive, physiological, biochemical and molecular aspects [8], such as yield and biomass reduction [9,10], stomatal closure to reduce water loss through transpiration, compromised CO_2_ availability [11], reduced photosynthetic performance, and chlorophyll degradation [12]. However, the water deficit technique has the potential to increase the post-harvest quality of lettuce, such as increasing phenolic and antioxidant content [13], and maximize water productivity and economic returns [14].

There are several substances used as biostimulants that act as plant stress attenuators, divided into organic substances, such as seaweed extracts, humic substances and hydrolyzed and inorganic substances, such as silicon (Si) [15]. The beneficial effects of Si application in many plant species are reported in the literature. The Si enhances production and fruit quality [16], promotes the biofortification of plants for human consumption [17], increases the availability and accumulation of some macro and micronutrients, and increases water-use efficiency [18]. The benefits are more evident in rice and wheat crops; in vegetables, the percentage found of this element in the biomass is lower, as it has a lower absorption capacity when compared to grasses, but there are also positive results in vegetables, especially in lettuce and tomato [18].

The strongest effects of Si application are observed in plants under abiotic or biotic stresses [18,19]. The improvement in the performance of plants under water deficit can be explained by the activity of enzymatic and non-enzymatic antioxidants and osmoprotective substances [20,21,22,23]. Si contributes to maintaining higher photosynthesis and transpiration and enhanced root water uptake [24]. The element improves the anatomical characteristics of leaf, maintains the ultrastructure of chloroplasts and protects the biological membranes [25,26], reduces water loss through the cuticle due to its improved thickness [27], and can prevent lipid peroxidation [17]. The beneficial roles of Si regarding other plant stresses are to promote mitigation of the negative effects caused under light stress conditions [28], and also to act against biotic agents in plants, releasing anti-pathogenic substances within plants in order to attenuate the incidence of infecting agents [29].

The high susceptibility of the lettuce crop to water stress ensures management options to maintain and even increase productivity under conditions of limited water availability. The integration of ecofriendly techniques such as deficit irrigation and biostimulant application is a sustainable strategy that could be studied in agricultural systems. Many studies show the positive results of Si application as a water deficit attenuator, but most of them use foliar application; it may be that the application via soil (fertigation) could be a viable alternative for the application of this beneficial element. Although Si-mediated mitigation of plant stress has been extensively investigated in different crops, especially cereal crops such as rice and wheat, limited information is available regarding the beneficial role of the soil application of Si in lettuce crops under water stress. It might be necessary to focus research on extensively cultivated vegetables to enhance comprehension and explore their potential value in horticulture. This study aims to determine the beneficial role of Si applied as a plant biostimulant through fertigation as a mitigator of water stress in lettuce crops and define a possible deficit irrigation strategy to increase water productivity in this crop.

## 2. Results

### 2.1. Crop Water Requirement

**Figure 1:**
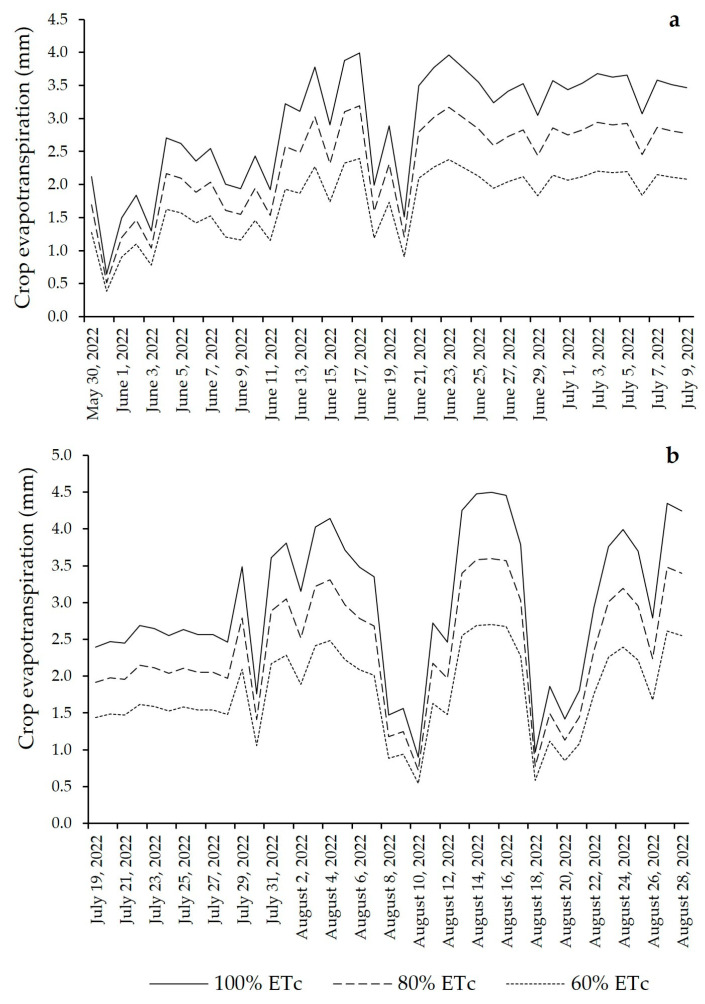
Crop evapotranspiration (mm) during the lettuce crop cycles: (**a**) first cycle and (**b**) second cycle.

The daily ETc calculated for the crop is shown in Figure 1. The total water demand of the lettuce crop for the two cycles was close; in the first cycle, the demand was 120.1 mm for 100% ETc, 96.1 mm for 80% ETc and 72.1 mm for 60% ETc. In the second cycle, the total water consumption was 122.4 mm, 97.9 mm and 73.4 mm for 100%, 80% and 60% ETc, respectively.

### 2.2. Yield and Plant Growth Parameters

**Figure 2:**
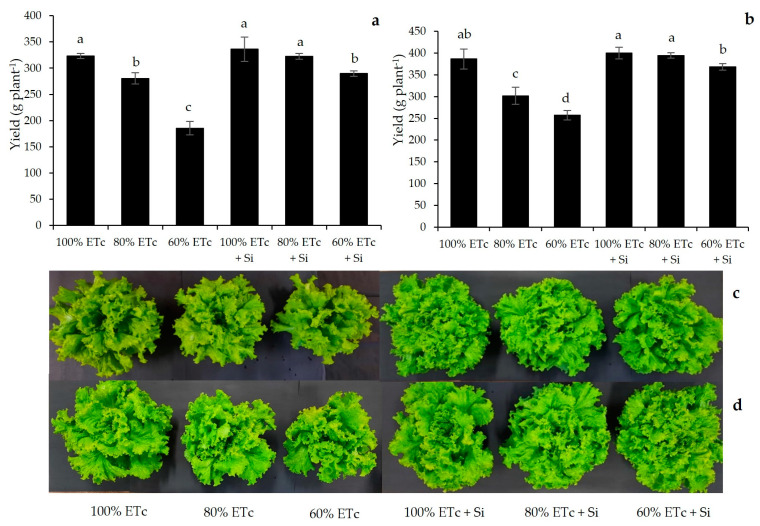
Lettuce yield (g plant^−1^) produced under different water replacement levels and silicon application for the two crop cycles: (**a**,**c**) first cycle; (**b**,**d**) second cycle. ETc: crop evapotranspiration; Si: silicon. Means (*n* = 20) indicated by different letters are statistically different by Tukey test at 5% significance. Interaction Si applications × irrigation levels: *p* < 0.001 for both cycles.

Deficit irrigation imposition during both lettuce crops caused a reduction in yield per plant (Figure 2a,b); the treatment with 60% ETc of water replacement level was the one that was most negatively affected. Compared to unstressed plants (100% ETc), these reductions were 42.6% and 33.50% for the first and second cycles, respectively. These effects were also observed in plant growth parameters, reducing shoot dry mass, total leaf area, number of leaves and root dry mass (Table 1). However, the interaction between silicon application and irrigation levels was significant (*p* < 0.05), increasing lettuce yield under water deficit, which can be easily visualized by the difference in plant size (Figure 2c,d).

The yield of the treatment with 80% ETc + Si was statistically similar to 100% ETc without Si in the first cycle and the treatment with 60% ETc + Si was similar to 100% ETc without Si in the second cycle. But, when analyzing the association of Si application in treatments with 100% ETc, there was no yield increase. In general, Si application was also effective in increasing the evaluated growth parameters. When compared, the two levels of water replacement with deficit irrigation (60% and 80% + Si) were superior in almost all these parameters evaluated in relation to the same treatments without Si application.

### 2.3. Photosynthetic Gas Exchange Parameters

Photosynthetic rate, stomatal conductance, intercellular CO_2_ concentration and transpiration rate were influenced by the irrigation levels (Table 2). Regardless of Si application, the treatment with 100% ETc showed higher values, while treatments with water stress were lower. However, this situation changed with Si application, where the photosynthetic rate values and the intercellular CO_2_ concentration of the treatment with 80% ETc + Si were statistically similar to 100% ETc without Si in both cycles.

In all the physiological parameters evaluated, the treatment with 60% + Si was equal to or superior to the treatment with 80% ETc without Si. When compared, both water replacement levels with deficit irrigation (60% and 80%) with Si application were similar regarding stomatal conductance and superior to the same treatments without Si application.

### 2.4. Chlorophyll and Carotenoid Measurements

The data in Table 3 show that deficit irrigation caused a reduction in all lettuce leaf pigments analyzed. Comparing the irrigation levels without Si application, for the first cycle, the decrease generated by deficit irrigation was 46.8% and 59% in Chl_a_, 50% and 61% in Chl_b_, 48.3% and 59.9% in Chl_a+b_, and 35.3% and 43.2% in carotenoids for 80% and 60% ETc, respectively, in relation to 100% ETc. The second cycle showed a similar trend of leaf pigment reduction from the imposed water stress. The two treatments with 100% ETc were the ones that presented the highest values of the three evaluated chlorophyll parameters. Unstressed plants (100% ETc) treated with Si showed no difference in the concentration of chlorophyll in leaves compared to untreated ones; however, under deficit irrigation, both treatments (80% and 60% ETc) with Si application increased in relation to the same ones without Si application.

### 2.5. Relative Water Content and Canopy Temperature

**Figure 3:**
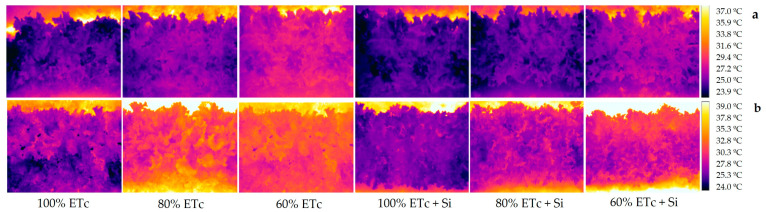
Thermal imaging of lettuce produced under different water replacement levels and silicon application for two crop cycles: (**a**) first cycle; (**b**) second cycle. ETc: crop evapotranspiration; Si: silicon.

Lettuce plants subjected to deficit irrigation presented a significant reduction in the RWC of lettuce leaves (Table 4). The treatments with 100% ETc presented the highest values, However, Si application improved the RWC of the plants under water-stress conditions. In the first cycle, the treatment of 80% ETc + Si increased 6.3%, and 60% ETc + Si increased 7.7% in relation to the same treatments without Si application. Thermal imaging was effective in detecting differences in the canopy temperature of lettuce; when analyzing Figure 3, it can be observed that treatments with 100% ETc were colder than the treatments with deficit irrigation. The canopy temperature of these plants presenting a darker color (purple) is lower compared to the treatments with water stress that presented a warmer color (yellow), showing that the canopy temperature was higher.

The average canopy temperature (Table 4) evidenced that both treatments with deficit irrigation (80% and 60% ETc) without Si application were the highest, with the average temperature for these treatments being 27.4 °C and 26.7 °C for the first cycle, and 30.6 °C and 31.2 °C for the second cycle, respectively. This corresponds to a temperature difference in relation to the treatment with 100% ETc of 1.5 °C and 2.2 °C for the first cycle and 3.6 °C and 3.1 °C for the second cycle, respectively. Although Si application has reduced these differences; in the first cycle, plants subjected to 80% ETc + Si were statistically similar to unstressed plants (100% ETc); moreover, in the second cycle, both treatments under water stress (80% and 60% ETc + Si) presented the same canopy temperature when compared to unstressed plants.

### 2.6. Water Productivity

**Figure 4:**
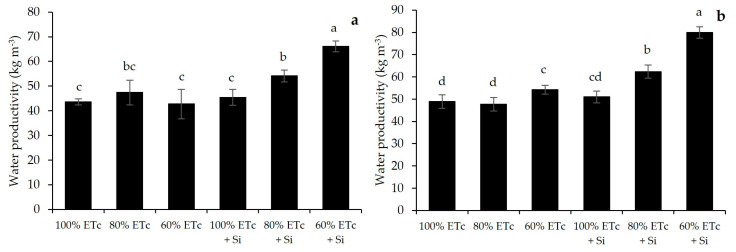
Water productivity (kg m^−3^) of lettuce produced under different water replacement levels and silicon application for two crop cycles: (**a**) first cycle; (**b**) second cycle. ETc: crop evapotranspiration; Si: silicon. Means (*n* = 20) indicated by different letters are statistically different by Tukey test at 5% significance. Interaction Si applications × irrigation levels: *p* < 0.001 for both cycles.

Figure 4 highlights that Si application significantly improved WP when submitted to deficit irrigation. The treatment with 60% ETc + Si had the highest WP in both lettuce cycles, followed by treatment with 80% ETc + Si. When compared to 100% ETc without Si, the treatment with 60% ETc + Si increased the WP by 51.6% in the first cycle and 61% in the second cycle. And when comparing the same treatment, with 80% ETc + Si, the WP increased by 24.1% and 27.5% in the first and second cycle, respectively.

### 2.7. Silicon Content Determination

The data in Table 5 reveal that Si application was significant (*p* < 0.05) only in terms of Si application, and there was no difference between the three treatments of irrigation level. The soil application of Si was superior to the control treatment, justified by the increase in Si concentration even in the soil and leaf in both cycles.

## 3. Discussion

This study explored the effects of Si application through fertigation under different water regimes (100%, 80% and 60% ETc) in two lettuce crop cycles. The results showed the significant potential of Si application to mitigate the negative effects caused by a water deficit in lettuce crops. The data showed positive increments in productive, morphological, physiological and nutritional aspects, as well as standing out as an alternative crop management to obtain favorable yields using less water in its production cycle, increasing water productivity and corroborating to create strategies for the water-use management of this leaf vegetable crop under subtropical conditions. The ability of plants subjected to silicon application to reduce the negative effects under this abiotic stress is complex and can be explained by some changes in metabolic pathways, in physiological and biochemical aspects.

It was observed that when subjected to water deficit conditions, there was a reduction in lettuce yield, and the greater the imposed stress, the greater the negative effects on the plants. Under these conditions, there was an impairment of gas exchange, reduction in transpiration rate, stomatal conductance and CO_2_ assimilation, and consequently inhibition of the proper functioning of photosynthesis. The decrease in photosynthesis is related to the reduction in the chlorophyll content of lettuce leaves under stress conditions [12]. Chlorophyll is important in the process of energy conversion in the plant as light energy captured by chlorophyll is used to convert water and CO_2_ into organic compounds such as glucose, essential for the energy production required for the growth and development of plants [30]. The reduction in the leaf area of plants under water stress can also be evidenced to reduce the light interception area and impact on photosynthesis. There is also an impact on biochemical aspects, such as the occurrence of oxidative damage, ROS production, and accumulation of proline and abscisic acid in the leaves [8].

When plants are under water stress, they seek to reduce the transpiration rate, closing their stomata to reduce water loss due to low availability in the soil; thus, leaf temperature tends to rise and be higher than air temperature. This phenomenon was detected by using the thermal camera, observing the difference in the color depending on the water regimes adopted. The thermal images of treatments with greater water availability (100% ETc) were darker, indicating that the canopy temperature was low compared to treatments with water stress, whose images showed that the canopy temperature was higher, and the RWC of the leaves support these results, whose values were higher in treatments that presented lower canopy temperature. This shows that these plants were in excellent turgidity conditions, with the plant cells being well hydrated and the internal pressure increasing, causing an increase in the rigidity and volume of the cells. This phenomenon is important for maintaining the plant’s structure and its ability to stand upright, as well as playing a crucial role in nutrient transport and photosynthesis [30].

It can be observed that Si application in treatments with water stress (especially in plants with 60% ETc + Si) was effective in maintaining lower canopy temperature and a higher RWC, compared to the same water-stress treatment without Si. This positive result is related to the role of silicon in regulating leaf transpiration and root water absorption [24], in which plants under these conditions were able to increase their root biomass and, consequently, had better conditions for water absorption, in which, after absorption, water is translocated through the xylem to the leaves and the transpiration process occurs. The results showed that even with stress, plants with silicon increased stomatal conductance and the transpiration rate, presenting a higher RWC and lower canopy T°C; as leaf turgidity increased, more water was used in their metabolic and physiological processes. This can also be explained by the maintenance of cell homeostasis, ameliorating the adverse effects of water stress in plants [8] and increasing antioxidant enzyme activity, photosynthetic pigments and osmoprotective substances [17,20,31]. The improvement in water balance in the Si application through fertigation may result from the increase in the thickness of the leaf cuticle, providing greater rigidity, and causing greater tolerance to stress [27]. In relation to improving conditions for increasing water absorption by increasing root biomass, it also can be associated with greater uptake and accumulation of macro and micronutrients in plants [32]. Therefore, for plants under water stress that had a reduction in biomass and leaf area, with Si application, they were able to develop better, achieving greater leaf area, number of leaves and total biomass.

It can be observed that the treatments with Si application had an increase in their Si content, so the increments in Si concentration were favorable to mitigate the negative effects of water stress and increase some morphological, productive and physiological parameters. Dou et al. [33] also had positive responses with the application of Si in tomato cultivation, increasing fruit yield and water productivity. Analyzing all the results, it is observed that water productivity in lettuce can be increased by using the water deficit associated with the application of a biostimulant-containing silicon. It was indicated that the treatment with 80% ETc + Si obtained similar productivity as 100% ETc without silicon in the two consecutive cycles of lettuce cultivation, showing that it is possible to reduce the volume of water by 20% without causing any damage to productivity, therefore increasing the water productivity for lettuce crop. Similar results were discussed by Farooq et al. [1] with physiological and agronomic approaches for improving water-use efficiency in crop plants.

Therefore, the action mechanism behind the benefits of Si under stress can be highlighted, such as stimulating the secondary metabolism of plants, and modulating the expression of genes involved in the production of secondary compounds, such as flavonoids and phytoalexins [29]; in this study, carotenoid increments were observed. In addition, Si application induces the production of antioxidant enzymes and proline, which are substances involved in the defense response of plants against abiotic stresses [20,22]. In this research, Si application favored the increase in the photosynthetic properties of plants under water deficit. Si can improve the efficiency of photosynthesis, increasing the rate of CO_2_ assimilation by plants, due to the increase in cell rigidity and the ability to keep leaves erect, favoring sun exposure and CO_2_ absorption, and it can also stimulate the production of chlorophyll [17,30]. Si applications were also beneficial when analyzing the nature of this beneficial element, acting as a physical and structural barrier, with the accumulation of silica in the leaf epidermis, maintaining high leaf water potential even under stress [22].

## 4. Materials and Methods

### 4.1. Local and Environmental Conditions

**Figure 5:**
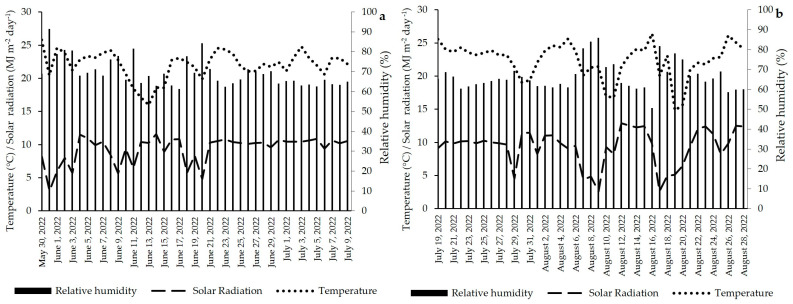
Average relative humidity (%), average temperature (°C) and total solar radiation (MJ m^−1^ day^−1^) inside the protected environment during the lettuce crop cycles: (**a**) First cycle and (**b**) Second cycle.

The experiment was conducted at the Biosystems Engineering department at University of São Paulo (22°42′ S, 47°37′ W and 546 m of altitude), Piracicaba, Brazil. The city’s climate according to Köppen is classified as humid subtropical zone (Cw) with 21.6 °C average temperature, 73% annual relative humidity and 1280 mm of annual precipitation [34]. Lettuce plants were grown in a protected environment of 20 m in length and 12 m in width under rain shelter conditions with a transparent plastic cover (diffuser film) 150 µm thick and 4.5 m ceiling height, surrounded by an anti-aphid nylon mesh. Lettuce cultivation was realized in two crop cycles; first cycle occurred from 30 May 2022 to 9 July 2022 and second cycle from 19 July 2022 to 28 August 2022. During the crop cycles, temperature, solar radiation and relative humidity were measured by a meteorological station with a CR23X datalogger (Campbell Scientific, Logan, UT, USA) (Figure 5) present in the center of the experimental area, under the transparent plastic cover. The soil of the experimental area is classified as Oxisols by Soil Taxonomy [35]. The physical and chemical characteristics of the soil are presented in Table 6 and Table 7, respectively.

### 4.2. Plant Material, Treatments and Experimental Design

The lettuce cultivar used was Vanda^®^, which has long leaves, a bright light green color and moderate crispness, presenting thick stem and vigorous root system. The experiment was conducted in a completely randomized design, with a factorial scheme (2 × 3), with three irrigation levels (60%, 80% and 100%) of crop evapotranspiration (ETc) and with and without silicon application. These three irrigation levels represent conditions of severe water stress, moderate water stress and optimal water conditions for lettuce crops, respectively. Five beds were built of 1.25 m length × 0.4 m width × 0.3 m depth per treatment, totaling thirty beds with a spacing of 1 m between them. Containing eight plants each, with spacing of 0.3 m between plants in the row, and 0.2 m between rows. The useful area consisted of the four central plants, totaling twenty repetitions per treatment. Lettuce seedlings were planted in the experimental area after 25 days of sowing,

The product Totale Silício^®^ was used as source of silicon. The product contains 750 g L^−1^ of SiO_2_ and 45 g L^−1^ of K_2_O, which is a fluid in suspension with a density of 1.20 g mL^−1^. The dose applied corresponds to 1.25 mL L^−1^, the product was applied three times during the crop cycle, at 10, 20 and 30 days after transplanting the seedlings (DAT), and each bed received a volume of 2 L per application through fertigation. Control treatment that did not receive any silicon application, received this volume with only water.

The deficit irrigation levels (60% and 80% ETc) were imposed with 5 DAT for seedling acclimatization. So, until this day, all treatments received 100% ETc. Deficit levels were calculated based on the 100% ETc, reducing 20% and 40% of the water volume to 80% and 60% ETc, respectively.

### 4.3. Crop Evapotranspiration Calculation

The ETc was obtained using the FAO 56 method from climate data obtained by the meteorological station installed inside the experimental area. The reference evapotranspiration (ETo) was estimated daily using the FAO 56 method by Penman–Monteith equation [36], with ETo (mm day^−1^) described by Equation (1):

(1) $$ETo=\frac{0.408\Delta \left(R_{n}-G\right)+\gamma \frac{900}{T+273}u_{2}(e_{s}-e_{a})}{\Delta +\gamma (1+0.34u_{2})}$$

where Δ represents the slope of the saturation vapor pressure–temperature relationship at mean air temperature (kPa °C^−1^), Rn is the net radiation at the crop surface (MJ m^−2^ d^−1^), G is the soil heat flux density (MJ m^−2^ d^−1^). T is mean daily air temperature (°C) and u_2_ is wind speed (m s^−1^), γ is the psychometric constant (kPa °C^−1^), e_s_ − e_a_ is the vapor pressure deficit computed from the saturation vapor pressure (e_s_, kPa) and the actual vapor pressure (e_a_, kPa).

The ETc (mm day^−1^) was calculated according to Equation (2), using the product of ETo multiplied by the crop coefficients (Kc) in relation to the phenological development of lettuce. The Kc values used were 0.70 (initial), 1.00 (intermediary) and 0.95 (final) [36].

(2) ETc=ETo×Kc

where ETo: reference evapotranspiration (mm day^−1^) and Kc: crop coefficient.

### 4.4. Irrigation and Fertigation

Irrigation and fertigation were realized using a drip irrigation system with self-compensating drippers spaced at 0.20 m, with a flow rate of 1.6 L h^−1^ and a service pressure of 1 bar, using an irrigation pump KSB^TM^ 500N (KSB SE & Co. KGaA, Frankenthal, Germany), Each bed was irrigated individually and controlled through the opening and closing of a hydraulic register installed at the beginning of each bed. A Top drip PC AS^®^ tape (NaanDan by Rivulis, Leme, RJ, Brazil) was inserted per bed, 1.25 m long, containing seven emitters self-compensating and anti-siphon, with a cascade labyrinth system that allows an efficient self-cleaning effect and provides excellent resistance to clogging, totaling a flow of 11.2 L h^−1^ per bed.

The irrigation system presented a uniform flow, representing 98.5% of Christiansen’s uniformity coefficient (CUC) and a distribution uniformity coefficient (CUD) of 96.8%. The quality parameters of the water used presented an apparent color < 0.2 (Hazen unit), conductivity of 138.1 µS cm^−1^, pH of 7.1, turbidity of 0.4 (turbidity unit), total CaCO_3_ of 46 mg L^−1^, calcium hardness of 32.3 mg L^−1^ and magnesium hardness of 13.7 mg L^−1^.

Non-deformed soil samples were collected for hydro-physical characterization of the soil at the beginning of the experiment; on the first day, irrigation was performed to add the water necessary to increase the soil water to field capacity level for the soil profile in all beds. In the next days, irrigation was performed daily using values obtained from ETc. Fertigation was carried out every seven days, with calcium nitrate as the main soluble source of nitrogen applied through the drip irrigation system, with dosage of 40 kg ha^−1^ N [37].

### 4.5. Yield and Plant Growth Parameters

The lettuce yield (g) was determined by the fresh mass of the aerial part for each plant individually. After harvesting, it was immediately weighed using semi-analytical balance (±0.001 g). Regarding plant growth parameters, the number of leaves, total leaf area (cm^2^), shoot dry mass (g), root fresh mass (g) and root dry mass (g) were analyzed.

The number of leaves was obtained by detaching the leaves from the stem and counting the leaves that were longer than 5 cm. After that, all these leaves were collected to estimate the total leaf area with a LI 3100 area meter (LI-COR™). The leaves, stems and roots were dried at 65 °C until reaching a constant weight, in order to obtain shoot (leaves + stem) and root dry mass using semi-analytical balance (±0.001 g). Roots were removed from the soil after harvesting the aerial part with the aid of a straight shovel 0.3 m deep, individually removing a block containing soil + root, and then carefully washing with water to completely remove the soil without damaging the roots.

### 4.6. Photosynthetic Gas Exchange Parameters

The photosynthetic gas exchange parameters of lettuce were measured from 9:00 to 11:00 in fully expanded intermediate leaves with solar radiation interception at 35 DAT for both cycles, when the plant was fully formed, the leaves were well developed and the plant had reached the desired size, 5 days before harvesting. The net photosynthetic rate (A, μmol m^−2^ s^−1^), stomatal conductance (gs, mol m^−2^ s^−1^), intercellular carbon dioxide concentration (Ci, μmol mol^−1^) and transpiration rate (E, mmol m^−2^ s^−1^) were all measured by a portable photosynthesis measurement system (LI 6400 XT, LI-COR Biosciences Company, Lincoln, NE, USA). The photosynthetic photon flux density (PPFD) adopted was 800 μmol m^−2^ s^−1^ and with 400 μmol mol^−1^ of CO_2_ concentration.

### 4.7. Chlorophyll and Carotenoid Measurements

Leaf pigment content was determined according to Lichtenthaler [38]. Chlorophyll _a_ (Chl_a_), chlorophyll _b_ (Chl_b_), total chlorophyll _a+b_ (Chl_a+b_) and carotenoid concentration were determined by spectrometry using acetone 100% as extractor. To extract the pigments, the leaf tissue sample (150 mg) was collected in fully expanded intermediate leaves at 34 DAT, and was submerged in 2 mL of pure acetone and kept at a dark and low temperature until the tissue was depigmented. The extract was placed in quartz cuvettes (2 mL) for absorbance determination in spectrophotometer (EvolutionTM 300, Thermo Fisher Scientific, Waltham, MA, USA). Absorbance was measured at 3 different wavelengths: 661.6, 644.8 and 470 nm, and the calculations were made based on Equations (3)–(6) and expressed as (µg g^−1^).

(3) $${Chl}_{a}=\left[\left(11.24\times A661.6\right)-\left(2.04\times A644.8\right)\right]$$

(4) $${Chl}_{b}=\left[\left(20.13\times A644.8\right)-\left(4.19\times A661.6\right)\right]$$

(5) $${Chl}_{a+b}=\left[\left(7.05\times A661.6\right)+\left(18.09\times A644.8\right)\right]$$

(6) $$Carotenoids=\left[\left(1000\times A470\right)-\left(1.9\times {Chl}_{a}\right)-\left(63.14\times {Chl}_{b}\right)\right]$$

### 4.8. Canopy Temperature

Canopy temperature was measured by using a FLIR T640 thermal camera model Duo^TM^ Pro R (Teledyne FLIR, Wilsonville, OR, USA) with emissivity (ε) set at 0.94. The images were obtained at a distance of 2 m of the crop canopy at around 11:00–12:00 a.m. on the same date as photosynthetic gas exchange evaluations, at 35 DAT. Thermal images were analyzed with the software program FLIR Thermal Studio^®^, version 2.0.11, analysis within the program was conducted by selecting an image area of 640 × 512 pixels from the center of each bed containing the lettuce plants. Average temperatures were obtained using the same software based on the temperature values of the selected pixels.

### 4.9. Relative Water Content

The relative water content (RWC) was estimated in intermediated lettuce leaves. Foliar segments (1 cm^2^) were cut and weighed to obtain fresh mass and were then submerged in distilled water to saturate for the next 24 h, in the dark, recording the turgor mass. The dry mass of the segments was obtained after 72 h at 65 °C. RWC was calculated by using Equation (7):

(7) $$WC\left(\%\right)=\left(\frac{\left(Mf-Md\right)}{\left(Mt-Md\right)}\right)\times 100$$

where Mf: fresh mass (g); Mt: turgor mass (g); and Md: dry mass (g).

### 4.10. Water Productivity

The water productivity (WP) was determined as the ratio of the fresh aerial part biomass of lettuce (yield) to the amount of total water applied by irrigation during each growing cycle, according to Equation (8), and expressed in (kg m^−3^).

(8) $$WP=\frac{Y}{IWA}$$

where Y: yield (kg) and IWA is the irrigation water applied (m^3^).

### 4.11. Silicon Content Determination

The determination of silicon content was carried out in plant tissue (leaf) and in the soil. Samples were collected at 35 DAT and were dried at 65 °C until reaching a constant weight for analysis. The Si content in the leaf was determined according to Silva [39], using NaOH (1%) as an extracting agent. The Si in the soil was determined according to the methodology described by Korndörfer et al. [40] and Pereira et al. [41], performing the extraction with CaCl_2_ 0.01 mol L**^−^**^1^. The contents (g kg**^−^**^1^) were determined by spectrometry at 660 nm and compared with a standard curve of Si standard solution.

### 4.12. Statistical Analyses

All data obtained from treatments were analyzed by using Sisvar software, version 5.6 [42]. Before performing the analysis of variance, the experimental data were subjected to the Shapiro–Wilk and Levene tests to verify residual normality and homoscedasticity, respectively. Data were submitted to two-factor analysis of variance (ANOVA) by F test at 5% of significance. After detecting that the interaction was significant, data were compared using Tukey’s test to separate into homogeneous groups.

## 5. Conclusions

In this research, deficit irrigation levels (60% and 80% of ETc) were associated with the application of a biostimulant that contains silicon, applied via fertigation as a strategy to mitigate water stress. It was concluded that deficit irrigation with silicon application is a sustainable strategy for lettuce production, aiming to save water resources, especially in the face of climate change, and helping to achieve global food security by improving water productivity and lettuce tolerance against water stress with a better growth, physiological, nutritional and yield response. After two crop cycles, it was found that favorable lettuce yields can be achieved using 20% less water during the crop cycle associated with silicon application.

## Figures and Tables

**Table 1:** Lettuce morphological parameters produced under different water replacement levels and silicon application for two crop cycles.

Silicon Application	Irrigation Levels (% ETc)	Shoot Dry Mass (g)	Total Leaf Area (cm^2^)	Number of Leaves	Root Dry Mass (g)
First cycle					
no Si	100	11.95 ^ab^	5013.95 ^ab^	19.40 ^a^	0.78 ^a^
	80	10.18 ^b^	4391.16 ^c^	17.60 ^b^	0.63 ^b^
	60	8.15 ^c^	3303.78 ^d^	14.20 ^c^	0.45 ^c^
+Si	100	12.73 ^a^	5380.94 ^a^	19.60 ^a^	0.80 ^a^
	80	11.72 ^ab^	5013.36 ^ab^	18.70 ^ab^	0.79 ^a^
	60	10.95 ^ab^	4679.76 ^bc^	17.90 ^b^	0.68 ^b^
Si		***	***	***	***
I		***	***	***	***
Si × I		*	*	***	***
Second cycle					
no Si	100	13.62 ^b^	5950.43 ^b^	20.50 ^ab^	0.88 ^a^
	80	10.63 ^c^	5042.28 ^c^	17.40 ^c^	0.74 ^b^
	60	9.37 ^c^	4023.31 ^d^	15.80 ^c^	0.54 ^c^
+Si	100	14.50 ^a^	6429.13 ^a^	22.40 ^a^	0.90 ^a^
	80	14.13 ^ab^	6165.06 ^ab^	21.00 ^ab^	0.87 ^a^
	60	13.34 ^b^	5945.76 ^b^	20.00 ^b^	0.75 ^b^
Si		***	***	***	***
I		***	***	***	***
Si × I		***	***	*	*

ETc: crop evapotranspiration; Si: silicon; I: Irrigation; CV: coefficient of variation. Different letters in the same column are statistically different by Tukey test at 5% significance for each crop cycle (*n* = 20). *** *p* < 0.001; * *p* < 0.05.

**Table 2:** Lettuce physiological parameters produced under different water replacement and silicon application for two crop cycles.

Silicon Application	Irrigation Levels (% ETc)	Photosynthetic Rate (μmol m^−2^ s ^−1^)	Stomatal Conductance (mol m ^−2^ s ^−1^)	Intercellular CO_2_ Concentration (μmol mol^−1^)	Transpiration Rate (mmol m^−2^ s ^−1^)
First cycle					
no Si	100	16.17 ^a^	0.41 ^b^	314.34 ^ab^	5.16 ^a^
	80	12.57 ^b^	0.32 ^cd^	294.11 ^bc^	3.69 ^b^
	60	10.30 ^c^	0.24 ^d^	280.75 ^c^	3.63 ^b^
+Si	100	17.73 ^a^	0.50 ^a^	336.38 ^a^	5.17 ^a^
	80	16.35 ^a^	0.42 ^b^	317.14 ^ab^	4.74 ^a^
	60	14.08 ^b^	0.38 ^b^	309.52 ^ab^	4.29 ^ab^
Si		***	***	***	**
I		***	***	***	***
Si × I		*	*	*	*
Second cycle					
no Si	100	16.56 ^b^	0.48 ^a^	305.14 ^b^	4.76 ^b^
	80	13.67 ^c^	0.33 ^c^	279.67 ^c^	3.65 ^d^
	60	10.88 ^d^	0.30 ^c^	270.56 ^c^	3.40 ^e^
+Si	100	19.64 ^a^	0.49 ^a^	333.38 ^a^	5.06 ^a^
	80	17.70 ^b^	0.39 ^b^	298.62 ^b^	4.22 ^c^
	60	15.21 ^bc^	0.37 ^b^	280.57 ^c^	3.84 ^d^
Si		***	*	***	***
I		***	***	***	***
Si × I		*	*	*	*

ETc: crop evapotranspiration; Si: silicon; I: Irrigation; CV: coefficient of variation. Different letters in the same column are statistically different by Tukey test at 5% significance for each crop cycle (*n* = 20). *** *p* < 0.001; ** *p* < 0.01; * *p* < 0.05.

**Table 3:** Lettuce leaf pigment parameters produced under different water replacement and silicon application for two crop cycles.

Silicon Application	Irrigation Levels (% ETc)	Chlorophyll a	Chlorophyll b	Total Chlorophyll	Carotenoids
		(µg g^−1^)			
First cycle					
no Si	100	7.44 ^a^	6.08 ^a^	13.53 ^a^	5.21 ^a^
	80	3.96 ^cd^	3.04 ^cd^	7.00 ^cd^	3.37 ^bc^
	60	3.05 ^d^	2.37 ^d^	5.42 ^d^	2.96 ^c^
+Si	100	7.91 ^a^	6.18 ^a^	14.09 ^a^	5.67 ^a^
	80	5.93 ^b^	4.58 ^b^	10.52 ^b^	3.97 ^b^
	60	4.92 ^bc^	3.86 ^bc^	8.78 ^bc^	3.98 ^b^
Si		***	***	***	***
I		***	***	***	***
Si × I		*	*	*	*
Second cycle					
no Si	100	5.93 ^a^	4.85 ^a^	10.79 ^a^	6.01 ^a^
	80	3.53 ^c^	2.81 ^c^	6.35 ^c^	3.56 ^cd^
	60	2.51 ^d^	2.07 ^c^	4.58 ^d^	2.78 ^d^
+Si	100	6.53 ^a^	5.15 ^a^	11.67 ^a^	6.43 ^a^
	80	4.76 ^b^	3.95 ^b^	8.71 ^b^	5.63 ^ab^
	60	4.36 ^b^	3.63 ^b^	7.99 ^b^	4.57 ^bc^
Si		***	***	***	***
I		***	***	***	***
Si × I		**	*	*	*

ETc: crop evapotranspiration; Si: silicon; I: Irrigation; CV: coefficient of variation. Different letters in the same column are statistically different by Tukey test at 5% significance for each crop cycle (*n* = 20). *** *p* < 0.001; ** *p* < 0.01; * *p* < 0.05.

**Table 4:** Relative water content and canopy temperature of lettuce produced under different water replacement and silicon application for two crop cycles.

Scheme	Irrigation Levels (% ETc)	Relative Water Content (%)	Canopy Temperature (°C)
First cycle			
no Si	100	83.66 ^a^	25.20 ^c^
	80	76.07 ^c^	26.68 ^ab^
	60	71.30 ^d^	27.40 ^a^
+Si	100	84.93 ^a^	24.70 ^c^
	80	80.84 ^b^	25.40 ^c^
	60	76.77 ^c^	26.23 ^b^
Si		***	***
I		***	***
Si × I		**	*
Second cycle			
no Si	100	85.96 ^a^	27.53 ^b^
	80	82.44 ^b^	30.62 ^a^
	60	74.94 ^c^	31.15 ^a^
+Si	100	86.54 ^a^	27.45 ^a^
	80	82.04 ^b^	28.35 ^b^
	60	79.83 ^b^	28.95 ^b^
Si		*	***
I		***	***
Si × I		**	**

ETc: crop evapotranspiration; Si: silicon; I: Irrigation; CV: coefficient of variation. Different letters in the same column are statistically different by Tukey test at 5% significance for each crop cycle (*n* = 20). *** *p* < 0.001; ** *p* < 0.01; * *p* < 0.05.

**Table 5:** Silicon concentration in soil and leaf of lettuce produced under different water replacement and silicon application for two crop cycles.

Silicon Application	Irrigation Levels (% ETc)	Si Concentration (g kg^−1^)	
		Soil	Leaf
First cycle			
no Si	-	4.10 ^b^	1.66 ^b^
+Si		4.49 ^a^	2.29 ^a^
Si		***	***
I		ns	ns
Si × I		ns	ns
Second cycle			
no Si	-	3.79 ^b^	1.30 ^b^
+Si		4.14 ^a^	1.67 ^a^
Si		***	***
I		ns	ns
Si × I		ns	ns

ETc: crop evapotranspiration; Si: silicon; I: irrigation; CV: coefficient of variation. Different letters in the same column are statistically different by Tukey test at 5% significance for each crop cycle (*n* = 20). *** *p* < 0.001; ns: not significant.

**Table 6:** Soil physical characteristics of the experimental area.

Layer (m)	Soil Density (g cm^−3^)	Moisture FC (m^3^ m^−3^)	Moisture PWP (m^3^ m^−3^)	Clay (%)	Silt (%)	Sand (%)
0–0.20	1.28	0.41	0.28	59.43	15.26	29.81
0.20–0.40	1.36	0.42	0.31	56.41	13.99	26.90

FC: field capacity; PWP: permanent wilting point.

**Table 7:** Soil chemical characteristics of the experimental area in 0–0.20 m soil layer.

Soil Characteristics	First Cycle	Second Cycle
pH CaCl_2_	5.8	5.6
Organic carbon (g dm^−3^)	26.0	23.0
Calcium (mmolc dm^−3^)	53.0	65.0
Magnesium (mmolc dm^−3^)	21.0	21.0
Potassium (mmolc dm^−3^)	7.8	8.2
Phosphorus (mg dm^−3^)	69.0	70.0
Sulfur (mg dm^−3^)	25.7	24.8
Aluminum (mmolc dm^−3^)	0	0
Hydrogen (mmolc dm^−3^)	14.0	16.0
Boron (mg dm^−3^)	0.2	0.2
Copper (mg dm^−3^)	1.2	1.5
Iron (mg dm^−3^)	9.0	8.0
Manganese (mg dm^−3^)	8.1	9.5
Zinc (mg dm^−3^)	2.7	2.6

## Data Availability

Data are contained within the article.
